# American Medical Association

**Published:** 1848-04

**Authors:** 


### AMERICAN MEDICAL ASSOCIATION.

By our exchanges we perceive that the various medical institutions of the country will be generally represented in the approaching meeting of the Association. The Counsellors of the Medical Society of Massachusetts have appointed *fifty* delegates. How many more will be sent by other bodies in that state we know not; but at that rate, throughout the country, the number of the whole will be great indeed. Several county societies in Pennsylvania have appointed their representatives, as have all the associations and institutions of Philadelphia. Of the names of all these we have not been informed, but the following we know to have been appointed:

*Jefferson Medical College*—Professors Huston and Pancoast.

*College of Physicians*—Drs. Hays, Jackson, Bond, Condie, Fox, A. Stillé, Pepper, King, J. R. Paul and C. D. Meigs.

